# Electromyographic Pattern during Gait Initiation Differentiates Yoga Practitioners among Physically Active Older Subjects

**DOI:** 10.3389/fnhum.2017.00300

**Published:** 2017-06-14

**Authors:** Thierry Lelard, Pierre-Louis Doutrellot, Abdou Temfemo, Said Ahmaidi

**Affiliations:** ^1^EA-3300: Adaptations Physiologiques à l’Exercice et Réadaptation à l’Effort, Faculté des Sciences du Sport, Université de Picardie Jules VerneAmiens, France; ^2^Service Medecine Physique et Rééducation, Centre Hospitalier UniversitaireAmiens, France; ^3^Department of Biological Sciences, Faculty of Medicine and Pharmaceutical Sciences, University of DoualaDouala, Cameroon

**Keywords:** anticipatory postural adjustments, gait, aged, yoga, electromyography

## Abstract

During gait initiation, postural adjustments are needed to deal with balance and movement. With aging, gait initiation changes and reflects functional degradation of frailty individuals. However, physical activities have demonstrated beneficial effects of daily motor tasks. The aim of our study was to compare center of pressure (COP) displacement and ankle muscle co-activation during gait initiation in two physically active groups: a group of walkers (*n* = 12; mean age ± SD 72.6 ± 3.2 years) and a yoga group (*n* = 11; 71.5 ± 3.8 years). COP trajectory and electromyography of leg muscles were recorded simultaneously during five successive trials of gait initiation. Our main finding was that yoga practitioners had slower COP displacements (*p* < 0.01) and lower leg muscles % of coactivation (*p* < 0.01) in comparison with walkers. These parameters which characterized gait initiation control were correlated (*r* = 0.76; *p* < 0.01). Our results emphasize that lengthy ankle muscle co-activation and COP path in gait initiation differentiate yoga practitioners among physically active subjects.

## Introduction

In older adults, physiological changes and physical inactivity are associated with a decrease in personal independence in general and in walking ability in particular. Indeed, older subjects with impaired gait are scared of falls and thus limit their physical activity and activities of daily living (Alexander, 1994; Maki and McIlroy, 1996). For example, individuals adopt a more conservative, basic gait pattern (Menz et al., 2003). Because of socio-economics impact of falling, several studies were lead to determine predictive factors of falling in older population. Gait initiation parameters (as described below) may be sensitive indicators of balance dysfunction and the risk of falls in older adults (Chang and Krebs, 1999). Indeed, gait initiation is a transient phase during which postural control and balance maintenance systems are highly active. Specifically, gait initiation consists in creating forward momentum from a quiet stance. Muscle activity creates internal forces that dissociate the center of pressure (COP) from the center of gravity (COG), in order to produce the initiation step (Mann et al., 1979; Brunt et al., 1991). While momentum creation disturbs balance, anticipatory postural adjustments (APA) are needed to deal with balance and allow efficient step initiation.

Several studies have evidenced the degradation of gait initiation with age. The older people show a decrease in the peak moment arm between the body’s COG and the COP (Brunt et al., 1991; Polcyn et al., 1998; Chang and Krebs, 1999). The decrease in this forward momentum is associated with lower COG velocity and lower first step amplitude (Patchay et al., 1997, 2002; Halliday et al., 1998). It has been suggested that this change in kinetic gait initiation parameters occurs because older subjects do not tolerate a deviation of the body as a whole (COG) from the ground reaction forces (COP). In order to initiate gait and compensate for impairments in the moment arm, older subjects have to perform additional trunk movements (Martin et al., 2002).

The observed changes in kinetic parameters have also been explained in terms of impaired muscle activation patterns. The role of muscle activity is to create internal forces in order to dissociate the COP from the COG (Mann et al., 1979; Brunt et al., 1991). For that reason, the electromyographic (EMG) sequence recorded during gait initiation can be considered as “a motor program that adjusts the configuration of external forces, which acts directly on the position of the COP and joint position” (Crenna and Frigo, 1991). Several studies described a stereotypical pattern of muscle activation in healthy, younger adults: (i) starting from a postural quiet stance, postural muscles are activated in order to maintain postural alignment; and (ii) in order to initiate gait *per se*, postural muscle activity is inhibited and motor muscles are then activated (Mann et al., 1979; Brunt et al., 1991, 1999). Difficulties in inhibiting antigravity muscles prior to movement are correlated with the age-related loss of Betz cells in the motor cortex (Scheibel et al., 1977; Scheibel, 1985). Higher levels of muscle coactivation have been reported in older subjects during activities of daily living–such as bipedal stance (Laughton et al., 2003; Nagai et al., 2011) and dynamic balance (Hortobágyi and DeVita, 2000; Larsen et al., 2008; Schmitz et al., 2009; Pereira and Goncalves, 2011). During gait initiation, the duration of postural and motor muscle coactivation increases with age. Indeed, it appears that the degradation of central mechanisms in gait initiation translates into a decrease in the ankle muscle antagonist coordination pattern (Polcyn et al., 1998; Henriksson and Hirschfeld, 2005), i.e., coactivation of the tibialis anterior (TA) and triceps surae muscles. The difference between young adults and older adults is more obvious for the swing limb (SWL), which shows greater degradation than the stance limb (STL) does (Polcyn et al., 1998).

It has often been suggested that physical activity improves functional ability in older people (Lord and Castell, 1994; Maki and McIlroy, 1996; Lelard and Ahmaidi, 2015). As an indicator of functional status, physically active olders show higher walking velocity compared to aged-matched sedentary adults. However, it seems that the characteristics of the physical activity, duration and frequency of training sessions induce differentiated effects on motor ability. Tai Chi training also showed an enhancement of gait initiation control (Hass et al., 2004; Vallabhajosula et al., 2014). These studies focused on CoP-CoG displacements during APA. These findings demonstrated that proprioceptive physical activity could help to improve gait initiation control via better COP displacement. Gait initiation is a pre-programmed task (Fiolkowski et al., 2002) which need APA controlled by an open-loop mechanism (Massion, 1992). As neuromuscular activity reflects motor program, previous results may explain this improvement by enhanced neuromuscular coordination after proprioceptive training (Chen et al., 2012a,b). Indeed, Nagai et al. (2012) have demonstrated significant changes in muscle coactivation after balance training (relative to an untrained group). Most of these studies reported beneficial effects of proprioceptive practice on postural control, however, differential effects depending on the type of practice were described on postural control (Gauchard et al., 2003). As described by these authors, proprioceptive activities such as yoga consist to produce a sequence of postures that need to deal with balance and movements improving body schema knowledge. To our knowledge, no study was performed to compare the effects of different types of physical activity on gait initiation.

Since differentiated effects on postural control were previously reported according the type of training program, we hypothesize that practicing different type of physical activity would show differences in APA during gait initiation. To initiate the first step, the central nervous system has to deal with the antigravity function of the posture and production of movements. Motor program has to switch from postural activity (Triceps Surae) to focal movements (TA). A time course analysis of co-activation and COP displacement might then be an effective indicator of efficiency of gait initiation. The aim of the present study was thus to investigate potentially differentiating patterns of leg muscle co-activation and COP displacements during gait initiation in yoga practitioners and physically active group (regular walkers).

## Materials and Methods

### Subjects

Twenty-three volunteers healthy subjects aged from 68 years to 78 years took part in the study. Regular yoga practitioners (*n* = 11; 71.5 ± 3.8 years old; 1.59 ± 0.08 m height; 66.8 ± 9.3 kg weight) and walkers subjects (*n* = 12; 72.6 ± 3.2 years old; 1.61 ± 0.03 m height; 64.3 ± 8.6 kg weight) were recruited from community dwelling. The group of yoga practitioners was standardized in terms of time (more than 1 year) weekly sessions (1) and sessions duration (1 h) of practice. The physically active group was selected on the basis of a questionnaire. Their physical activity level was equal or higher than 1 h of walking per week. All subjects signed prior the study an informed consent which was accepted by the local ethical committee (Comité de protection des Personnes Nord Ouest 2, Amiens, France) in accordance with Helsinki Declaration of 1975. Prior to taking part in the study, all participants reported their medical history. Clinical examination and questioning was conducted in order to exclude subjects showing any possible causes of balance alteration (medication or disease). Subjects were free from any disease which could influence postural maintaining and were able to walk without external help. Subjects did not show cognitive impairments with a score superior to 24 points in Mini-Mental State Evaluation (Folstein et al., 1975). The participants were independent as revealed by a maximal score in the Activities of Daily Living (Katz et al., 1963).

### Experimental Devices

A Piezoelectic force plate (Kistler type 9281 B11, Kistler AG, Winterthur, Switzerland) associated with a calculator was used to assess the temporal time-course of the COP displacements in antero-posterior (AP) and medio-lateral (ML) from the ground reaction forces and their moments in the three planes. The analogic signal was digitized at a sampling frequency of 1000 Hz.

Electromyograms (EMG) of leg muscles were collected using bipolar Ag/AgCl surface electrodes (Beckmann, 8-mm diameter) on the first leg that leaves the floor (initial SW limb). Before the electrode positioning, the skin was slightly abraded and cleaned with an alcohol solution in order to reduce the interelectrode resistance to below 5 kΩ. An electrolytic gel was placed between the skin and the electrodes to insure electrical contact. The electrodes were fixed (2 cm apart center to center) over the muscle bellies for TA and lateral gastrocnemius (LG) muscles. The EMG signals were amplified and filtered using a bandwidth of 10–1000 Hz (Gould 6600), and then addressed to an analog-digital converter piloted by the Turbolab software (SM2I, France) for their digitization at a sampling rate of 1000 Hz.

### Procedure

After two learning trials, the subjects performed five gait initiation trials. In order to standardize the starting conditions, the subjects placed their bare feet on foot marks drawn on the platform. They had to keep their arms by their sides, relax their jaw and fix a spot on a wall 10 m in front. The experiments took place in a quiet room with no visual perturbations.

In each test, the experimenter triggered the acquisition of the COP displacement and EMG signals. The order to initiate gait was given by the experimenter after at least 2 s of steady-state postural recording (judged by visual inspection of the EMG signal).

### Data Analysis

Usually, based on COP displacement, three gait initiation phases were described (Halliday et al., 1998; Martin et al., 2002; Hass et al., 2004), the first phase represents the backward COP displacements that create the forward momentum thanks to COP-COG dissociation, the second the weight bearing transfer from SWL to STL, and the third the forward COP displacement.

**Figure 1:**
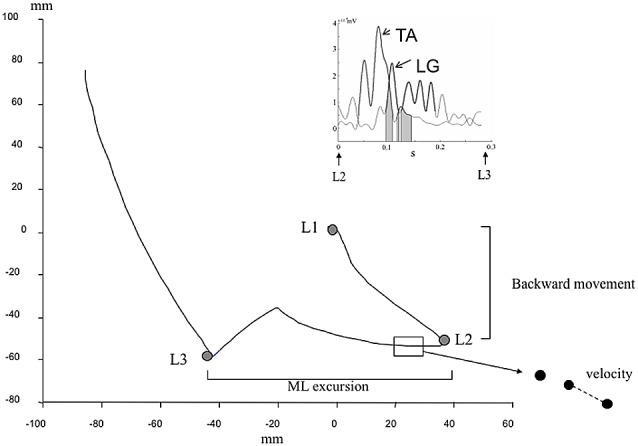
Kinetic and electromyographic (EMG) data used to describe gait initiation. Center of pressure (COP) displacement during gait initiation. L1 represents the position of the COP in normal quiet standing. L2 represents the backward of COP. L3 represents the COP displacement toward the stance limb (STL). Parameters used were the backward COP position during the phase 1 (L1–L2), the medio-lateral (ML) excursion, the velocity (path reported to phase duration). Insert represents tibialis anterior (TA) and lateral gastrocnemius (LG) vs. time during the weight bearing transfer from swing limb (SWL) to STL (From L2 to L3). The bold lines represent when muscle are activated (>25 ms consecutive above the threshold corresponding to the mean ± 3 standard deviation). Gray area represents the phase of coactivation of LG and TA muscles.

These phases were identified in our study thanks to the gait initiation events using landmarks on the COP trajectory (Figure 1). Landmark 1 (L1) represents the mean position of COP during quiet stance. Landmark 2 (L2) represents the most posterior and lateral position toward the SWL of COP location. Landmark 3 (L3) represents the most posterior and lateral position toward the STL of COP location. The end of the recording of COP displacement represents the outgoing of COP from the force plate. The identification of these landmarks was conducted with an application of Matlab Program (The Mathworks, Inc., Natick, MA, USA), under visual control. In order to characterize the COP displacement, we calculated the excursion separating two landmarks, the average velocity (sum of distance separating two points of acquisition related to time elapsed in the phase (Figure 1).

For EMG data, we have analyzed the period corresponding to weight bearing transfer from STL to SWL during which an inhibition of postural muscle and activation of motor muscles were reported. From an algorithm developed with Matlab program, we determined phasic activation of LG and TA of the SWL under visual control. The Matlab application was developed using previous methodological studies (Mickelborough et al., 2004). EMG data were smoothed with low-pass filter cut off frequency of 20 Hz. The threshold for EMG activation detection was defined as mean added with 3 standard deviations of reference phase. The muscle was considered as active after 25 ms of consecutive above the threshold. Then, we calculated relative time of coactivation of LG and TA muscles during weight bearing transfer from SWL to STL (ratio of time of coactivation with time elapse in the phase).

### Statistics

Statistical analysis was carried out with Statview software (SAS Institute, Cary, NC, USA). Given that the data were normally distributed (according to a Kolmogorov-Smirnov test) and the equality of variance Levene median test) was confirmed, an unpaired *t*-test was used to compare the physically active group and yoga group in terms of kinetic and EMG values. The threshold for statistical significance was set to *p* < 0.05.

We also assessed the relationship between kinetic COP displacement parameters and muscle coactivation by applying Pearson’s correlation test.

## Results

### COP Movements

**Figure 2:**
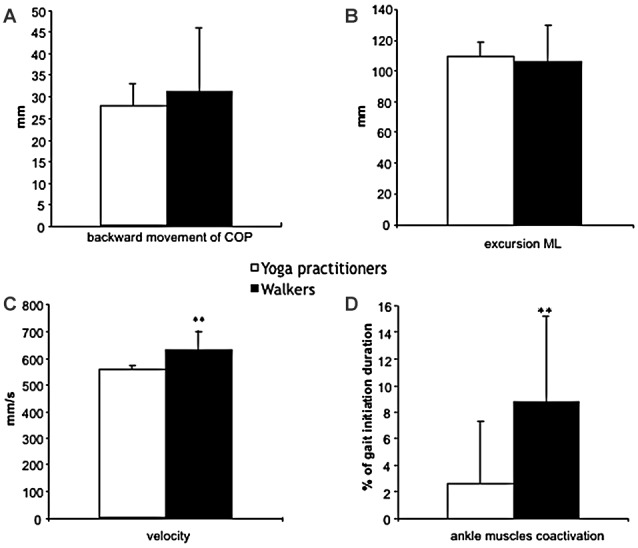
Kinetic and EMG data recorded in yoga (□) and in walkers (■) subjects (mean and standard deviation). **(A)** The backward movement of COP during the phase 1 of gait initiation. **(B)** The excursion of COP displacement (in mm) during phase 2 of gait initiation. **(C)** Velocity of COP displacement (in mm) during phase 2 of gait initiation. **(D)** Percent of time during which LG and TA are coactivated during phase 2 of gait initiation. Significant differences were expressed as **p < 0.01.

The yoga and physically active groups do not differ significantly in terms of displacements during the first phase of gait initiation (Figure 2A). The mean ± SD backward movement of COP position shows non-significant lower values in the yoga group than in the group of walkers (−27.8 ± 5.3 vs. −31.1 ± 13.6 mm respectively).

For the second phase of gait initiation, we characterized the excursion and velocity of COP displacement from the SWL to the STL. We did not find significant differences in COP excursion during this phase (110.8 ± 12.1 vs. 102.5 ± 19.7 mm for the yoga and physically active groups, respectively; Figure 2B). The COP velocity was significantly lower in yoga practitioners than in the walkers (555.2 ± 32.5 vs. 624.0 ± 50.3 mm.s^−1^, respectively; *p* < 0.01; Figure 2C).

### Leg Muscle Co-Activation

During gait initiation, we found significant differences in leg muscle co-activation between the two physically active groups (Figure 2D). In fact, leg muscle co-activation is lower in yoga practitioners than in the walkers (3.9 ± 6.0% vs. 10.5 ± 5.1%, respectively; *p* < 0.01).

The relationships between kinetic variables and leg muscle co-activation reveal that muscle co-activation is correlated with the velocity measured during the SWL-to-STL phase (*r* = 0.76; *p* < 0.01).

## Discussion

The objective of the present study was to assess the effect of regular yoga practice in gait initiation control in the elderly. This was characterized in terms of COP excursion, COP velocity and muscle co-activation. We hypothesized that Yoga practitioners present differences in APA during gait initiation compared to physically active older adults. Indeed, Gauchard et al. (2003) demonstrated that proprioceptive activities enhance sensory inputs and improve the subject’s knowledge of the body scheme and gravity effects. Significant results were reported during the SWL to STL transfer for the COP velocity and % of leg muscle coactivation between the two groups. Lastly, the observed link between COP path and muscle co-activation duration confirmed the interaction between muscle co-activation and the COP trajectory during gait initiation.

During gait initiation, the central postural control system has two main functions: (i) maintenance of balance; and (ii) creation of the anteroposterior moment needed to produce the first step (via a stereotyped pattern of leg muscle activation). In the present study, we did not observe any significant intergroup differences in the rearward COP excursion during the first phase of gait initiation. Previous studies have found that older and disabled subjects exhibit a lower arm peak moment (i.e., a smaller COP-COG distance; Polcyn et al., 1998; Chang and Krebs, 1999; Martin et al., 2002). Based on COP displacements, the present study does not allow to demonstrate differentiated effect between type of physical activity practiced on COP rearward during the first phase of APA.

The second phase of gait initiation consisted in transferring body weight from the initial SWL to initial STL. In the present study, we reported higher average velocity of the COP in the physically active group as compared to the yoga group for a similar ML excursion. In previous studies, smoother COP displacement during gait initiation was seen after 48 weeks of tai chi practice (Hass et al., 2004) and was also described in healthy older subjects (relative to disabled subjects; Martin et al., 2002). The reason for this significant difference remains unclear: the lower COP velocity in the yoga group might be related to the specific, slow movements performed during this activity. Then, it might be the consequence of a behavioral change in yoga practitioners.

However, our results might also be related to adaptations of the central nervous system in the yoga group (relative to the physically active group). To our knowledge, the present study is the first investigation conducted to explore differentiated coactivation of the leg muscles during gait initiation in two types of physical activity practitioners. The time course analysis of EMG data assessed the time during which muscles were involved in postural maintaining (LG) and movement (TA) (Polcyn et al., 1998; Henriksson and Hirschfeld, 2005). The duration of coactivation (expressed as a % of APA duration) might be an indicator of CNS adaptation to deal with postural constraints induced by gravity and movement production during the preparation phase of gait. This muscle pattern (inhibition of postural muscle) was described in healthy young adults (Mann et al., 1979; Brunt et al., 1991, 1999) and seem to be altered with aging and/or disability (Scheibel et al., 1977; Scheibel, 1985; Polcyn et al., 1998; Henriksson and Hirschfeld, 2005). In the present study, Yoga practitioners present lower TA/LG muscles coactivation than the physically active group. The result should be related to a previous study that demonstrated that muscle activation during gait initiation can reflect different behavior in counteracting and using gravity (Honeine et al., 2014).

One specific feature of the present study was the observed association between COP displacement and the EMG motor program. As previously described, the EMG sequence recorded during gait initiation can be considered as a motor program that acts on the COP’s position (Crenna and Frigo, 1991). APA are pre-programmed by an open-loop mechanism, then their improvements need better body scheme knowledge. In our study, we reported a significant correlation (*r* = 0.76; *p* < 0.01) between TA/LG coactivation and COP displacement. The relationship obtained between muscle co-activation (time of coactivation/time) and velocity (path/time), demonstrates that the path length is greater when the duration of muscle co-activation is longer. Leg muscle coactivation previously reported in frailty adults might also reflect hesitation or difficulties in initiating gait (i.e., pain, motor impairment, fear of falls, etc.). Several studies have shown that changes in ankle control occur with age and may be associated with an increased risk of falling. A previous study has shown that ankle dorsiflexion was significantly delayed and its amplitude was lower for fallers during gait (Kemoun et al., 2002). Simoneau et al. (2007) explained the decrease in dorsiflexion maximal voluntary contraction torque with aging by neural factor (Simoneau et al., 2007). The question of the benefit of reducing muscle co-activation in frailty adults should then be explored.

Our results suggest that the neuro-adaptation related to yoga performance can modify leg muscle pattern during gait initiation. This result should also be related to the reduction in muscle coactivation during other dynamic tasks after practice (Chu et al., 2009; Chen et al., 2012a,b; Nagai et al., 2012).

However, the present study may have some limitations inherent to cross-sectional studies. Even if our inclusion criteria allow us to select a healthy elderly population in this cross-sectional study, the two groups might not present similar health conditions. Comparison with other groups should help to understand the effects of physical activity on gait initiation by comparing our results with groups of young adults, sedentary older adults or with other type of physical activity (exercises at a fast pace way requiring balance). We can also note that due to the absence of kinematic data, we could not determine the position of the COG. Therefore, the COP-COG dissociation could not be assessed in the present study.

## Conclusion

The actual results obtained did not accurately demonstrated that gait initiation is more efficient (in terms of COP-COG dissociation and in term of amplitude of the first step) in yoga practitioners than in regular walkers. However, Yoga practitioners showed slower COP displacement associated with lower leg muscle coactivation. This last point result might be a marker of the use of a different strategy to prepare the first step. Further studies are needed to explore the clinical interests of proprioceptive activities on muscle coactivation during gait initiation and the relationship between gait initiation efficiency (COP-COG, step length) and leg muscle coactivation.

## Author Contributions

TL, P-LD and SA: project creation. TL: data collection. TL, P-LD and AT: data analysis. TL, P-LD, AT and SA: discussed the results; revised the manuscript. TL and AT: drafted the manuscript.

## Conflict of Interest Statement

The authors declare that the research was conducted in the absence of any commercial or financial relationships that could be construed as a potential conflict of interest.
